# Exosomes promote cetuximab resistance via the PTEN/Akt pathway in colon cancer cells

**DOI:** 10.1590/1414-431X20176472

**Published:** 2017-11-13

**Authors:** S. Zhang, Y. Zhang, J. Qu, X. Che, Y. Fan, K. Hou, T. Guo, G. Deng, N. Song, C. Li, X. Wan, X. Qu, Y. Liu

**Affiliations:** 1 Department of Medical Oncology, the First Hospital of China Medical University, Shenyang, China; 2 Key Laboratory of Anticancer Drugs and Biotherapy of Liaoning Province, the First Hospital of China Medical University, Shenyang, China

**Keywords:** Cetuximab, Exosome, PTEN, Akt, Colon cancer

## Abstract

Cetuximab is widely used in patients with metastatic colon cancer expressing wildtype KRAS. However, acquired drug resistance limits its clinical efficacy. Exosomes are nanosized vesicles secreted by various cell types. Tumor cell-derived exosomes participate in many biological processes, including tumor invasion, metastasis, and drug resistance. In this study, exosomes derived from cetuximab-resistant RKO colon cancer cells induced cetuximab resistance in cetuximab-sensitive Caco-2 cells. Meanwhile, exosomes from RKO and Caco-2 cells showed different levels of phosphatase and tensin homolog (PTEN) and phosphor-Akt. Furthermore, reduced PTEN and increased phosphorylated Akt levels were found in Caco-2 cells after exposure to RKO cell-derived exosomes. Moreover, an Akt inhibitor prevented RKO cell-derived exosome-induced drug resistance in Caco-2 cells. These findings provide novel evidence that exosomes derived from cetuximab-resistant cells could induce cetuximab resistance in cetuximab-sensitive cells, by downregulating PTEN and increasing phosphorylated Akt levels.

## Introduction

Colon cancer is the third most commonly diagnosed malignancy worldwide (1). Radical surgery is an effective therapeutic approach for non-metastatic colon cancer. However, the prognosis of patients with metastatic colon cancer remains very poor. Cetuximab (C225), a chimeric human-mouse anti-epidermal growth factor receptor (EGFR) monoclonal antibody, can improve clinical outcomes in some patients with metastatic colon cancer expressing wild type KRAS. However, only a subgroup of individuals with KRAS wildtype cancer benefit from C225 treatment, suggesting the existence of other drug resistance mechanisms, in addition to KRAS gene mutation (2,3). Recent studies indicated that primary and acquired drug resistance induced by aberrant mutations in oncogenes or tumor suppressor genes could reduce C225 efficacy in some patients (4–7). Additionally, tumor cell heterogeneity and alteration of the tumor microenvironment were shown to contribute to C225 resistance (8,9). Thus, cell-cell communication within the tumor microenvironment is another potential mechanism of acquired drug resistance. Nevertheless, how drug resistance information is exchanged between cells is largely unclear.

Exosomes, also known as intraluminal vesicles of multivesicular bodies, are nanosized (30–150 nm) cup-shaped vesicles containing various proteins, lipids, and nucleic acids (10). They currently attract increasing attention in various research fields. Multiple studies have shown that numerous cell types, including tumor cells, secrete exosomes. In the field of oncology, exosomes were shown not only to have immune functions (11), but also to promote tumor metastasis, angiogenesis, and drug resistance. Recent studies assessing different types of cancers revealed that exosomes induce drug resistance by exporting drugs from cells and delivering resistance signals as well as neutralizing antibodies (12). Exosomes are also potentially important in colon cancer, but few studies of drug resistance have focused specifically on monoclonal agents. Therefore, it is important to explore the mechanism by which exosomes derived from colon cancer cells regulate cetuximab sensitivity.

In this study, we assessed the effects of exosomes derived from cetuximab-resistant RKO cells on cetuximab-sensitive Caco-2 recipient cells.

## Material and Methods

### Material

Cetuximab was purchased from Merck KGaA (Germany). LY294002 was purchased from Sigma (USA). Antibodies targeting EGFR, phosphor-EGFR (Tyr1068), Akt, phosphor-Akt (Ser473), PTEN, PI3-kinase and calreticulin were obtained from Cell Signaling Technology (USA). Anti-flotillin-1 was from BD Biosciences Pharmingen (USA), and anti-CD63 from Abcam (USA). Anti-GAPDH was purchased from Santa Cruz Biotechnology (USA).

### Cell culture

RKO and Caco-2 colon cancer cell lines were obtained from the Cell Bank of the Chinese Academy of Sciences (China). Both cell lines were grown in RPMI-1640 (Gibco-BRL, USA) supplemented with 10% fetal bovine serum (FBS), penicillin (100 U/mL) and streptomycin (100 mg/mL), in a humid atmosphere containing 5% CO_2_ at 37°C. Exosome-free FBS was prepared by centrifugation of FBS at 100,000 *g* for 14 h at 4°C.

### Exosome isolation and transmission electron microscopy

Cell supernatants were collected from RKO and Caco-2 cells after 48 h of culture in exosome-free conditioned medium, and treated with ExoQuick-TC (System Biosciences, USA) according to the manufacturer's instructions. Exosomes were collected by centrifugation, with the pellet resuspended in 100 µL phosphate buffered saline (PBS) and stored at −80°C until use. The protein content of exosomes was quantified with BCA Protein Assay Kit (Thermo Scientific, USA). Exosome specimens were fixed with 1% glutaraldehyde in PBS, and a 20 µL drop of each sample was placed on a carbon-containing grid and incubated for 1 min at room temperature for electron microscopy. Then, 20 µL of 2% phosphotungstic acid was used to stain each sample for 2 min, followed by observation under a JEM-1200EX electron microscope (JEOL, Japan).

### Exosome labeling

Exosome pellets were resuspended in 200 µL PBS containing 1 µL Vybrant DiD (Invitrogen, USA) for 30 min, followed by centrifugation at 4°C, 100,000 *g* for 2 h. The pellets were then resuspended in PBS and incubated with Caco-2 cells for 12 h. Fluorescence microscopy was used to image exosomes internalized by Caco-2 cells.

### MTT assay

The effects of various treatments on cell viability were assessed by the MTT assay. First, cells were seeded at 3000–4000 cells/well on 96-well plates and allowed to attach in culture medium supplemented with 10% exosome-free FBS. Cells were then co-cultured with exosomes at various densities for 48 h. Afterwards, the conditioned medium containing exosomes was removed, and cells were exposed to cetuximab for another 48 h. Next, 20 µL MTT solution (5 mg/mL) was added to each well and incubated for 4 h at 37°C. After incubation, the medium was carefully removed and the formazan crystals were dissolved in 200 µL dimethyl sulfoxide (DMSO). Absorbance at 570 nm was measured on a microplate reader (Model 550, Bio-Rad Laboratories, USA).

### Western blotting

Cells were washed three times with ice-cold PBS and resuspended in 1% Triton X-100 lysis buffer on ice, followed by protein quantification by the Lowry method. Equal amounts of total protein were separated by SDS-PAGE and transferred onto PVDF membranes (PerkinElmer, USA). The membranes were incubated with appropriate primary antibodies at 4°C overnight after blocking for 2 h at room temperature with 5% skimmed milk in trimethyl benzene sulfonyl tetrazole buffer (TBST). After three washes with TBST, the membranes were incubated with secondary antibodies for 30 min at room temperature. Finally, the immunoreactive protein bands were visualized with enhanced chemiluminescence reagent (SuperSignal Western Pico Chemiluminescent Substrate, USA), followed by imaging on an Electrophoresis Gel Imaging Analysis System (DNR Bio-Imaging Systems, Israel).

### Statistical analysis

Data are reported as means±SD. Student's *t*-test or one-way ANOVA followed by Dunnett's and Tukey's *post hoc* tests were used to evaluate differences between or among groups. SPSS 17.0 (SPSS Inc., USA) was used for the analyses. P<0.05 was considered statistically significant. Each experiment was repeated at least three times.

## Results

### Identification of exosomes derived from cetuximab-resistant RKO cells

**Figure 1.**
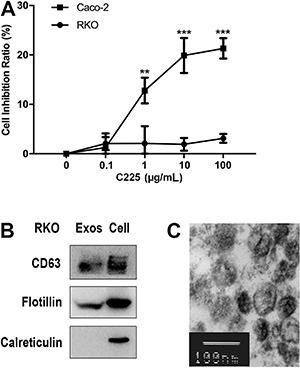
Identification of cetuximab-resistant RKO cell-derived exosomes. *A*, RKO and Caco-2 cells treated with increasing cetuximab concentrations (0, 0.1, 1, 10, and 100 μg/mL) for 48 h were assessed by the MTT assay to detect inhibition rates. Data are reported as means±SD. **P<0.01, ***P<0.001, compared to control (one-way ANOVA, with Dunnett's *post hoc* test). *B*, Western blot for detecting CD63, flotillin and calreticulin protein levels in exosomes from RKO cells and cell lysates with equivalent protein amounts. *C*, Transmission electron microscopy showing 30–100 nm size, saucer-shaped exosomes, enclosed by a lipid bilayer.

The inhibition rates of different C225 concentrations (1, 10, and 100 μg/mL) were significantly elevated in Caco-2 cells but not in RKO cells compared with the C225 control group (0 μg/mL; Figure 1A). These results indicated that RKO cells were resistant to cetuximab, unlike Caco-2 cells, which were cetuximab-sensitive. Next, exosomes were isolated from the cell supernatants of RKO cells previously cultured for 48 h in exosome-free medium. Western blotting was used to assess the exosome biomarkers CD63 and flotillin as well as the negative control marker calreticulin (Figure 1B). The presence of exosomes was confirmed by transmission electron microscopy and images indicated that exosomes derived from RKO cells were 30–100 nm in diameter, saucer-shaped, and enclosed by a lipid bilayer (Figure 1C).

### Induction of cetuximab resistance in Caco-2 cells by RKO cell-derived exosomes

**Figure 2.**
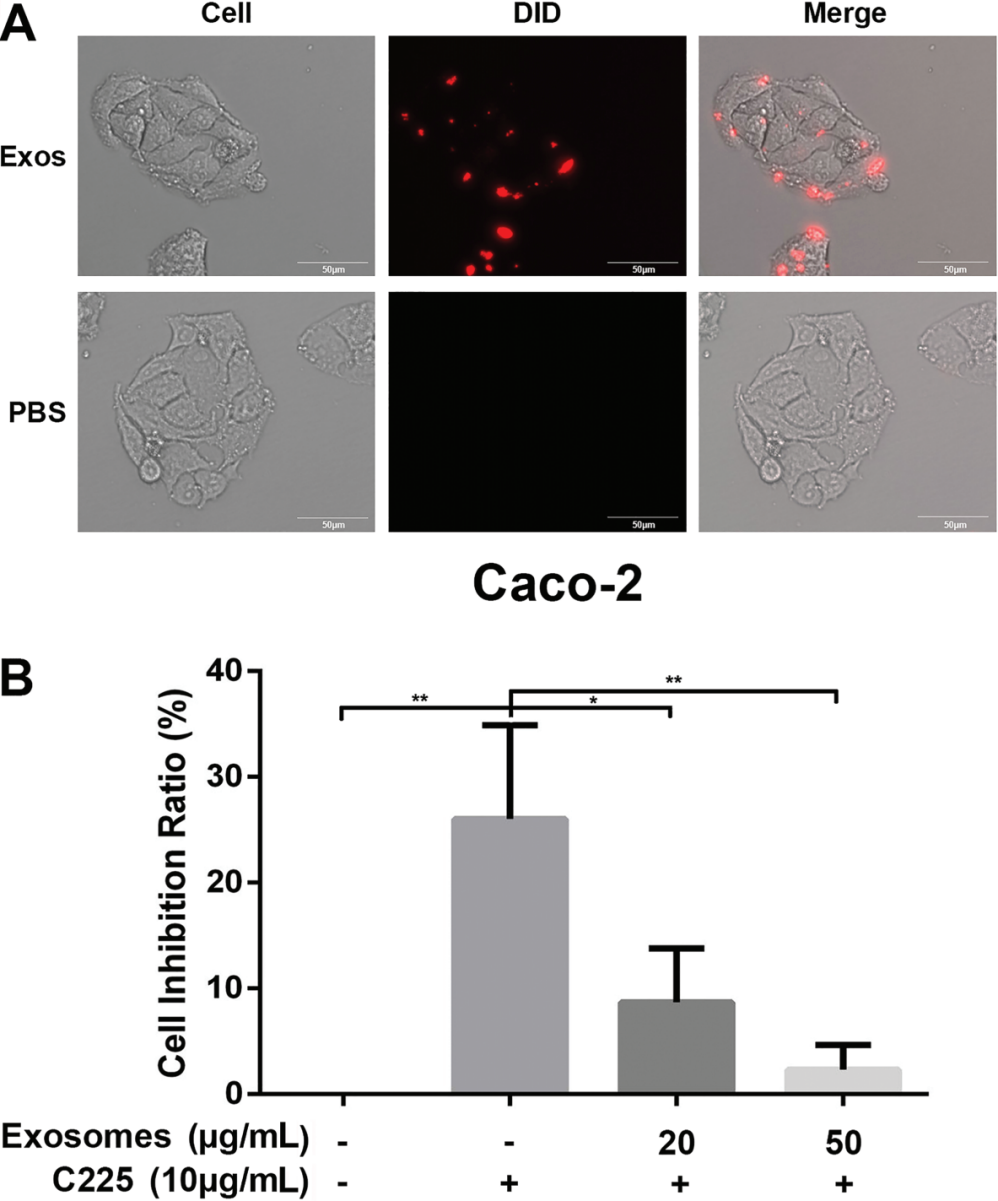
RKO cell-derived exosomes induced cetuximab resistance in Caco-2 cells. *A*, RKO-derived exosomes were labeled by DiD for 30 min followed by centrifugation at 100,000 *g* for 2 h (4^o^C) and co-culture with recipient Caco-2 cells for 12 h (PBS as control). Then, fluorescence microscopy imaging confirmed that exosomes were internalized by Caco-2 cells. Original magnification: 200×. *B*, The MTT assay was used to evaluate the effects of RKO-derived exosomes (20 and 50 µg/mL) on Caco-2 cell viability. Exosome-induced cetuximab (10 µg/mL) resistance was observed in Caco-2 cells. Data are reported as means±SD. *P<0.05, **P<0.01 (one-way ANOVA, with Tukey's *post hoc* test).

Fluorescence microscopy revealed that RKO cell-derived exosomes were internalized by Caco-2 cells (Figure 2A). To assess effects of exosomes derived from cetuximab-resistant RKO cells on Caco-2 cells, the latter were co-cultured with RKO cell-derived exosomes (20 and 50 µg/mL) for 48 h. Then, the exosome-loaded culture medium was removed, and cells were treated with cetuximab (10 µg/mL) for another 48 h. The cell inhibition rate was reduced from 26±8.89 to 8±5.13 and 2±2.31% after treatment with 20 and 50 µg/mL exosomes, respectively. Thus, Caco-2 cell viability in the presence of cetuximab was increased by co-culture with exosomes, indicating that RKO-derived exosomes induced drug resistance in cetuximab-sensitive cells (Figure 2B).

### Exosome-treated Caco-2 cells showed reduced phosphatase and tensin homolog (PTEN) levels and increased phosphor-Akt amounts

**Figure 3.**
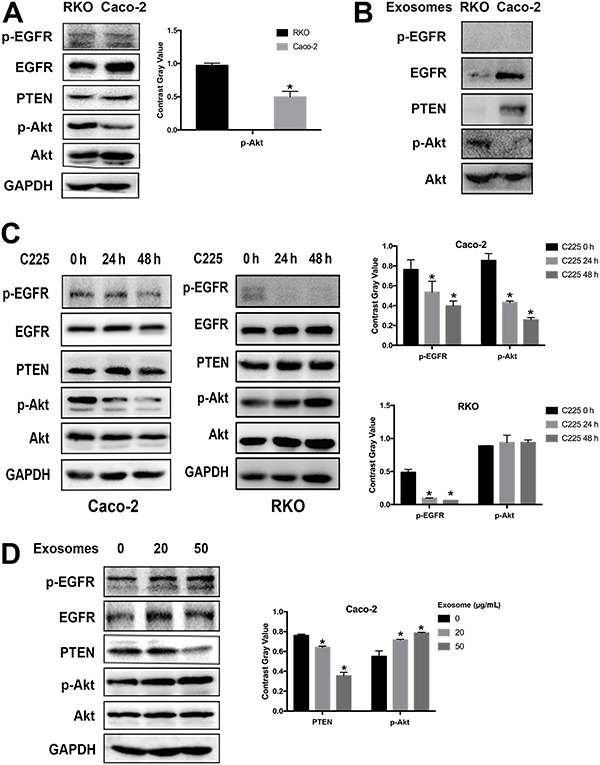
Exosome-treated Caco-2 cells showed reduced PTEN and increased phosphor-Akt amounts. *A*, *B*, Western blot was used to assess the expression levels of p-EGFR, EGFR, PTEN p-Akt, Akt and GAPDH in RKO, Caco-2 cells and their derived exosomes. Data are reported as means±SD. *P<0.05 (Student's *t*-test). *C*, Western blot revealed reduced p-EGFR and p-Akt protein levels, after treatment of Caco-2 and RKO cells with cetuximab (10 µg/mL) for 24 and 48 h, respectively. *D*, Western blot showed reduced PTEN levels and phosphor-Akt activation after Caco-2 cell co-culture with increasing amounts of RKO-derived exosomes (20 and 50 µg/mL). Data are reported as means±SD. *P<0.05, compared to control (one-way ANOVA, with Dunnett's *post hoc* test).

The mechanism by which cetuximab induces resistance in Caco-2 cells via RKO cell-derived exosomes is unclear. We previously showed that exosomes derived from gastric cancer cells facilitate recipient cell proliferation via the PI3K/Akt pathway (13). To assess whether PI3K/Akt signaling was also involved in the decreased cetuximab sensitivity observed in exosome treated Caco-2 cells, western blot was used to analyze the main components of this pathway. The basal levels of phosphor-Akt were different in Caco-2 and RKO cells (Figure 3A). In agreement, PTEN levels were also higher in exosomes derived from Caco-2 cells than in those from RKO cells (Figure 3B). Meanwhile, Caco-2 cell treatment with cetuximab for 48 h resulted in decreased EGFR and Akt phosphorylation levels. In contrast, treatment of RKO cells with cetuximab resulted in decreased EGFR phosphorylation but increased phosphor-Akt levels, indicating that Akt was the key effector involved in RKO cell drug resistance (Figure 3C). Co-culture of Caco-2 cells with RKO cell-derived exosomes (20 or 50 µg/mL) for 48 h resulted in decreased PTEN levels and increased Akt phosphorylation (Figure 3D). These findings suggested that RKO-derived exosomes decreased cetuximab sensitivity in Caco-2 cells through PTEN downregulation and Akt phosphorylation.

### RKO-derived exosomes induced cetuximab resistance in Caco-2 cells via the PTEN/Akt pathway

**Figure 4.**
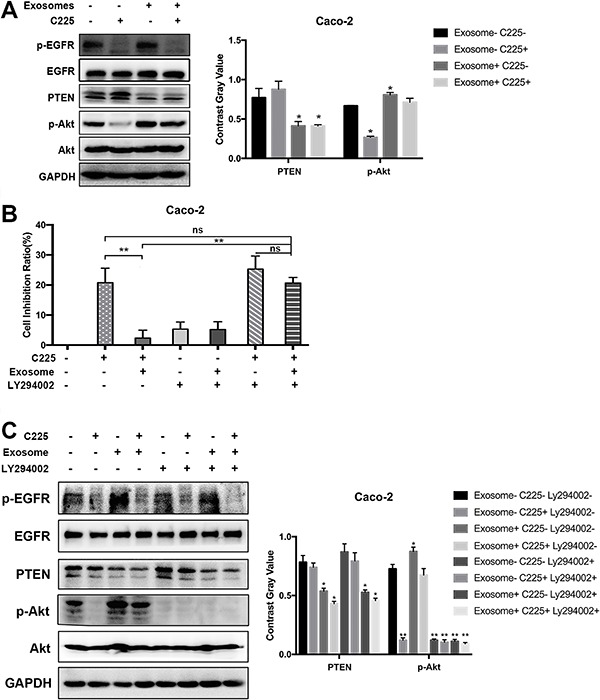
RKO-derived exosomes induced cetuximab resistance in Caco-2 cells via the PTEN/Akt pathway. *A*, Western blot was used to detect the expression levels of p-EGFR, EGFR, p-Akt, Akt, PTEN, and GAPDH in Caco-2 cells treated with cetuximab (10 μg/mL) for 48 h after pre-treatment with RKO-derived exosomes (50 μg/mL) for 48 h. *B*, The MTT assay showed that the Akt inhibitor LY294002 reversed the RKO-derived exosome induced C225 drug resistance in Caco-2 cells. Data are reported as means±SD. *P<0.05, **P<0.01 (one-way ANOVA, with Tukey *post hoc* test). *C*, Western blot was used to assess the changes of p-EGFR, EGFR, PTEN, phosphor-Akt, and Akt expression levels in Caco-2 cells treated with the Akt inhibitor LY294002. Data are reported as means±SD. *P<0.05, **P<0.01, compared to control (one-way ANOVA, with Dunnett's *post hoc* test).

PTEN expression was reduced while phosphor-Akt levels were increased after treatment with cetuximab (Figure 4A). To further confirm these findings, the Akt inhibitor LY294002 was added to Caco-2 cells co-treated with RKO cell-derived exosomes and cetuximab. The inhibition rate after cetuximab treatment was increased from 2±2.61 to 21±1.82% by LY294002, similar to that observed in Caco-2 cells treated with cetuximab alone (21±4.80%; Figure 4B). These findings demonstrated that cetuximab resistance induced in Caco-2 cells by RKO cell-derived exosomes was prevented by LY294002. Western blot showed that Akt phosphorylation was suppressed by LY294002 in cells treated with cetuximab and exosomes. These findings confirmed an important role for PTEN/Akt signaling in exosome-mediated cetuximab resistance (Figure 4C).

## Discussion

Cetuximab is widely used to treat metastatic colon cancer, but drug resistance limits its clinical use. Mutation of pivotal genes was established as a mechanism of drug resistance. However, other possible causes of cetuximab resistance have been proposed, including the tumor microenvironment and cellular interactions (8,9,14). Exosomes attract increasing interest, and the biological functions of exosome-mediated exchange of drug resistance information between homologous or heterogeneous cells have been explored (15). Among studies assessing colon cancer cell lines, Ragusa et al. (16) found that exosomes derived from Caco-2 (KRAS wildtype) cells pretreated with cetuximab promote the viability of HCT-116 (KRAS mutant) cells. In this study, we explored the mechanisms by which exosomes transfer drug resistance information from cetuximab-resistant to cetuximab-sensitive cells.

Because of the heterogeneity of tumors, we first confirmed that KRAS wildtype expressing RKO and Caco-2 cells had different sensitivities to cetuximab. We next demonstrated that RKO cell-derived exosomes induced drug resistance in cetuximab-sensitive Caco-2 cells. Following cetuximab treatment, EGFR and Akt phosphorylation levels were decreased in Caco-2 cells. In contrast, PTEN downregulation and increased Akt phosphorylation were observed in Caco-2 cells after co-culture with RKO cell-derived exosomes. To assess whether PTEN/Akt signaling was involved in the exosome-mediated drug resistance observed in Caco-2 cells, the effects of combined treatment with exosomes and cetuximab were evaluated. These exosomes downregulated PTEN and prevented cetuximab related reduction of phosphor-Akt levels. A previous retrospective study showed that PTEN deficiency predicts cetuximab resistance in metastatic colon cancer (17). This indicated that exosome-induced PTEN deletion might be a mechanism of cetuximab resistance.

Tumor-derived exosomes participate in many processes, including immune responses, metastasis, drug resistance, and drug delivery (15). Tumor-derived exosomes alter drug sensitivity of recipient cells by transferring proteins, mRNAs or micro RNAs (miRNAs). Multiple studies have investigated the contents of exosomes interacting with recipient cells (18,19). Wei et al. (20) found that exosomes derived from tamoxifen-resistant MCF-7 breast cancer cells induce tamoxifen resistance in tamoxifen-sensitive MCF-7 cells by delivering miR221/222. Furthermore, Challagundla et al. (21), and Ji et al. (22) showed that drug resistance could be induced not only by tumor cell-derived exosomes, but also by those derived from normal cells, via miRNA transfer. In this study, PTEN expression was lower in exosomes derived from RKO cells than in those obtained from Caco-2 cells. Because miRNAs are crucial to post-transcriptional modifications, the low PTEN levels observed in exosome-treated Caco-2 cells might result from regulation by miRNAs delivered by RKO cell-derived exosomes. Indeed, miRNAs are frequently mentioned as exosomal cargos that negatively regulate target proteins. Recently, Lasda et al. (23) demonstrated that circular RNAs are removed from cells by vesicle secretion. In addition, Qu et al. (24) found that exosomes induce sunitinib resistance in renal cancer cells by transmitting the long non-coding RNA lncARSR. These findings suggest that the functional cargos in exosomes require further investigation.

This study also revealed different PTEN levels in exosomes derived from RKO and Caco-2 cells, which was not the case for parental cells. This demonstrated that exosome origin is another potential cause of decreased PTEN levels in RKO cell-derived exosomes. Exosome composition is determined by multiple biological processes and other factors. Sun et al. (25) found that exosomes derived from TP53 mutant HCT116 cells are smaller than those obtained from TP53 wild type HCT116 cells. In addition to the oncogene status of parental cells, hypoxia, low pH, oxidative stress and other stress conditions can also affect exosome composition and secretion (26–28). Since RKO and Caco-2 cells have distinct genetic characteristics, such as BRAF and PIK3CA mutations and microsatellite instability (29), oncogene status is a potential cause of differences in PTEN and phosphor-Akt levels among their respective exosomes. Recently, extensive proteomic analysis of exosomes was performed to identify predictive and prognostic cancer biomarkers (30–32). Assessment of proteomic differences between cetuximab resistant and sensitive cells might identify pivotal markers of drug resistance and help elucidate the mechanisms underlying cetuximab resistance.

In conclusion, this study revealed a novel mechanism by which cetuximab induces resistance, involving exosomes derived from cetuximab-resistant colon cancer cells. Additionally, altering PTEN/Akt signaling in cetuximab-sensitive cells was crucial to exosome-mediated drug resistance. These findings suggested a new potential approach for reversing cetuximab resistance in wildtype KRAS colon cancer. Further research should focus on differences between exosomes derived from drug resistant cells and those from sensitive cells, to identify new candidate biomarkers for predicting cetuximab effectiveness.
